# Loss-of-activity-mutation in the cardiac chloride-bicarbonate exchanger AE3 causes short QT syndrome

**DOI:** 10.1038/s41467-017-01630-0

**Published:** 2017-11-22

**Authors:** Kasper Thorsen, Vibeke S. Dam, Kasper Kjaer-Sorensen, Lisbeth N. Pedersen, V. Arvydas Skeberdis, Jonas Jurevičius, Rimantas Treinys, Ida M. B. S. Petersen, Morten S. Nielsen, Claus Oxvig, J. Preben Morth, Vladimir V. Matchkov, Christian Aalkjær, Henning Bundgaard, Henrik K. Jensen

**Affiliations:** 1 Department of Molecular Medicine, Aarhus University Hospital, Palle Juul-Jensens Boulevard 99, DK-8200 Aarhus N, Denmark; 2 Department of Biomedicine, Aarhus University, Ole Worms Alle 4, DK-8000 Aarhus C, Denmark; 3 Department of Molecular Biology and Genetics, Aarhus University, Gustav Wieds Vej 10, DK-8000 Aarhus C, Denmark; 4 Institute of Cardiology, Lithuanian University of Health Sciences, Sukilėlių pr. 15, LT-50162 Kaunas, Lithuania; 5 Department of Biomedical Sciences, University of Copenhagen, Blegdamsvej 3, DK-2200 Copenhagen N, Denmark; 6 Norwegian Center of Molecular Medicine, Nordic EMBL Partnership, University of Oslo, Postboks 1137, Blindern, NO-0318 Oslo, Norway; 7 Institute for Experimental Medical Research, Oslo University Hospital, Postboks 4956 Nydalen, NO-0424 Oslo, Norway; 8 Unit for Inherited Cardiovascular Diseases, The Heart Centre, National University Hospital, Rigshospitalet, Copenhagen Health Science Partners, University of Copenhagen, Blegdamsvej 9, DK-2100 Copenhagen Ø, Denmark; 9 Department of Cardiology, Aarhus University Hospital, Palle Juul-Jensens Boulevard 99, DK-8200 Aarhus N, Denmark; 10 Department of Clinical Medicine, Health, Aarhus University, Palle Juul-Jensens Boulevard 82, DK-8200 Aarhus N, Denmark

## Abstract

Patients with short QT syndrome (SQTS) may present with syncope, ventricular fibrillation or sudden cardiac death. Six SQTS susceptibility genes, encoding cation channels, explain <25% of SQTS cases. Here we identify a missense mutation in the anion exchanger (AE3)-encoding *SLC4A3* gene in two unrelated families with SQTS. The mutation causes reduced surface expression of AE3 and reduced membrane bicarbonate transport. *Slc4a3* knockdown in zebrafish causes increased cardiac pH_i_, short QTc, and reduced systolic duration, which is rescued by wildtype but not mutated *SLC4A3*. Mechanistic analyses suggest that an increase in pH_i_ and decrease in [Cl^−^]_i_ shortened the action potential duration. However, other mechanisms may also play a role. Altered anion transport represents a mechanism for development of arrhythmia and may provide new therapeutic possibilities.

## Abstract (web summary)

Mutations in potassium and calcium channel genes have been associated with cardiac arrhythmias. Here, Jensen et al. show that an anion transporter chloride-bicarbonate exchanger AE3 is also responsible for the genetically-induced mechanism of cardiac arrhythmia, suggesting new therapeutic targets for this disease

## Introduction

The short QT syndrome (SQTS), first recognized in 2000^1^, is a rare, genetically determined, severe arrhythmogenic disease with a high risk of syncope, atrial and ventricular fibrillation, and sudden cardiac death (SCD)^2^. Cardiac events have a clear male preponderance, typically presenting during the 2nd or 3rd decade^3^. The treatment after aborted SCD and malignant ventricular arrhythmia is implantable cardioverter defibrillator (ICD) implantation^4^. SQTS has only been reported to present in an autosomal inherited dominant form. The diagnostic criteria for SQTS proposed by Gollob et al.^4^ were based on a combination of shortened duration of the repolarisation (QT interval), family- and clinical history. A frequency corrected QT (QTc) < 370 ms was suggested as an initial cutoff for diagnostic consideration of SQTS, whereas the HRS/EHRA/APHRS Expert Consensus Statement^5^ suggested QTc < 330 ms as a stand-alone criterion for the SQTS diagnosis or a possible diagnosis if QTc < 360 ms in combination with a pathogenic mutation, family- or clinical history.

Disease-causing mutations have been identified in the cardiac potassium ion channels *KCNH2* ^6,7^, *KCNQ1* ^8^ and *KCNJ2* ^9^ leading to gain of channel function and causing action potential duration (APD) shortening and elevated risk of cardiac arrhythmia. Loss-of-function mutations in genes coding for the subunits of the cardiac L-type calcium channels *CACNA1C*, *CACNB2* and *CACNA2D1*, have recently been identified as separate genetic causes of SQTS^10,11^. So far, only these six cation channels have been implicated in SQTS, and most expanded genetic screening therefore focuses on cation channel genes. However, considering that identified cation channel mutations explain <25% of SQTS cases^4^, other classes of genes are likely to be associated with the development of this disease. Such genes may encode anion channels, transporters, or proteins involved in pH regulation, which could all potentially alter the QT interval. A role for changes in pH is supported by the finding that lowering intracellular pH (pH_i_) prolongs QT interval^12^; a role for Cl^−^ is supported by the demonstration that modulation of Cl^−^ conductance in cardiomyocytes also changes APD^13^.

In this study, we identify a novel genetic etiology for SQTS in a family with presumed autosomal dominant inheritance of SQTS. We find that affected family members have a mutation in the anion exchanger Solute Carrier Family 4 Member 3 (*SLC4A3*) gene, which encodes a Cl,HCO_3_-exchanger (AE3). The mutation leads to a trafficking defect, decreased Cl,HCO_3_-exchange over the cell membrane, increased pH_i_ and it reduces QT-duration in zebrafish embryos. These findings represent a previously unappreciated mechanism for the development of SQTS.

## Results

### Cardiac manifestations in probands and relatives with SQTS

**Fig. 1:**
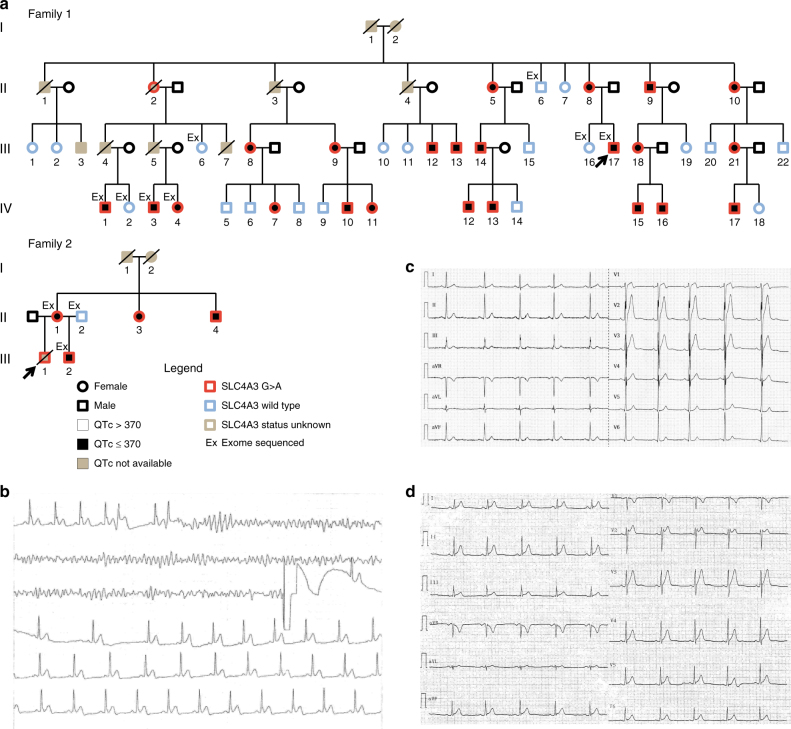
Pedigrees and representative ECG recordings in two families with SQTS. **a** Pedigree of family 1 and 2. Proband marked with an arrow. **b** ECG recording showing ventricular fibrillation and DC shock conversion to sinus rhythm in the proband in family 1. **c** ECG-12 from the proband in family 1. **d** ECG-12 from patient III-2 in family 2

Pedigrees and demographics for the families are presented in Fig. 1a and Supplementary Table 1. The index patient (III-17) in family 1 presented 31 years old with cardiac arrest during sleep. He was successfully resuscitated and admitted to hospital where a new episode of ventricular fibrillation was converted to sinus rhythm by DC shock (Fig. 1b). In his ECG, a QTc duration of 320 ms and a J-point to T-peak interval (Jp-Tp) of 120 ms (Fig. 1c) were noted. No other cardiac abnormalities were observed. A structurally normal heart was found in all relatives, except for subject IV-13, who showed dilated cardiomyopathy with decreased systolic left ventricular ejection fraction (LVEF) of 40%. This patient was later diagnosed with Limb-Girdle muscle dystrophy type 2I^14^. Four relatives (II-8, II-10, IV-1, III-13) experienced syncopes in the second decade, and two relatives (III-4, III-5) died suddenly and unexplained at the age of 41 and 42 years, respectively. In family 2, the proband (II-1) died suddenly unexpectedly at rest 22 years old. His brother (II-2) was asymptomatic with a structurally normal heart and a QTc of 320 ms and a Jp-Tp < 120 ms (Fig. 1d). The parents were asymptomatic; the mother’s QTc was 355 ms. Cascade screening in family 2 revealed two more asymptomatic mutation-carrying relatives (II-2, II-3) with short QTc’s in both (Fig. 1d and Supplementary Table 1). All family members are of European descent.

### Whole exome sequencing of family members

Initial analysis of the family 1 index patient using a targeted Next Generation Sequencing (NGS) gene panel including the SQTS genes, known to be associated with SQTS at that time (*CACNA1C*, *CACNB2*, *KCNH2*, *KCNQ1*, and *KCNJ2*, Supplementary Table 2) revealed no pathogenic mutations. Exome sequencing of four SQTS-affected individuals from family 1 (III-17, IV-1, IV-3, IV-4) along with four asymptomatic family members with normal ECGs (I-6, II-6, III-16, IV-2) was performed (Fig. 1a). Three members from family 2, who were referred to the clinic at the time of the analysis, were also exome sequenced (Supplementary Table 3).

Variants were filtered to include variants shared by all six SQTS individuals but not present in any of the five healthy controls. After removal of variants observed in publicly available databases (frequency > 5%) only 11 variants remained. Nine were outside gene regions, one was intronic (chr19:52149499:c421–11A > G, *SIGLEC14*), not predicted to affect splicing, and one was a heterozygous missense variant (chr2:220497051:c.1109 G > A NM_201574, p.R370H) in *SLC4A3* (Supplementary Table 3). The latter variant was not present in any of the publically available variant databases. The resulting amino acid change from arginine to histidine (R370H) is in the conserved E_367_-T-A-R-W-I-K-F-E-E_376_ motif of the SLC4 family. The variant was predicted as possibly damaging by PolyPhen2^15^ and had a CADD score 28.1 also indicating a deleterious effect of the variant^16^.

### ECG changes and manifestations in carriers and non-carriers

Cascade screening in family 1 identified a total of 23 carriers of the *SLC4A3* c.1109 G > A variant. All carriers had a QTc ≤ 370 ms (mean 340 ± 18 ms) and 19 non-carriers had a QTc > 370 ms (mean 402 ± 24 ms) (*p* < 0.001, Students two-tailed *t*-test) (Fig. 1a and Table 1). Post mortem analysis based on paraffin-embedded tissue showed that subject II-2 also carried the c.1109 G > A mutation, which makes the two relatively young SCD victims (III-4 and 5) obligate carriers of the c.1109 G > A variant. All *SLC4A3* c.1109 G > A carriers had Jp-Tp ≤ 140 ms (mean 121 ± 15 ms) compared to mean 173 ± 22 ms in non-variant carriers (*p* < 0.001, Students two-tailed *t*-test). In the unrelated smaller family 2, the four mutation-carrying relatives had QTc < 370 ms. Only men and no children experienced clinical symptoms. There were no differences in QTc (*p* = 0.78, Students two-tailed *t*-test) or Jp-Tp (*p* = 0.97, Students two-tailed t-test) between male and female carriers of the mutation (Table 1). Due to the clinical course in these two families with SCD and aborted SCD in the index patients and serious cardiac syncope in another relative (Fig. 1a and Supplementary Table 1) mutation carriers were offered implantation of a primary prophylactic ICD. After thorough information, 16 out of 25 (64%) of the asymptomatic mutation-carrying relatives, accepted ICD implantation. After a follow-up of a mean of 49 ± 9 month none of the mutation-carrying relatives including the aSCD survivor had experienced ICD therapy. In addition, the mutation-carrying relatives without an ICD were also asymptomatic.

**Table 1:** Electrocardiographic findings in *SLC4A3* c.1109 G > A mutation carriers and non-carriers from a large family with short QT syndrome

	*SLC4A3* c.1109 G > A (Mut)	*SLC4A3* Wild-type (WT)	*p*-value
All individuals (Mut *n *= 23; WT *n* = 19)			
QT (ms)	317 ± 25	378 ± 27	7.61E-09
QTc (ms)	340 ± 18	402 ± 24	2.13E-10
Jp-Tp (ms)	121 ± 15	173 ± 22	3.35E-09
Males (Mut *n *= 13; WT *n* = 9)			
QT (ms)	323 ± 24	364 ± 27	5.39E-03
QTc (ms)	341 ± 19	391 ± 15	3.59E-06
Jp-Tp (ms)	121 ± 13	162 ± 16	2.65E-05
Females (Mut *n *= 10; WT *n *= 10)			
QT (ms)	308 ± 23	391 ± 20	3.83E-07
QTc (ms)	339 ± 17	413 ± 26	4.57E-06
Jp-Tp (ms)	121 ± 18	182 ± 24	1.97E-05

*Jp-Tp* Jpoint-Tpeak interval, *QT* QT interval, *QTc* Bazett corrected QT interval

Data are presented as mean ± standard deviation

*P*-values determined by un-paired Students two-tailed t-test with Bonferroni’s correction

### Reduced membrane localization of the mutated AE3

**Fig. 2:**
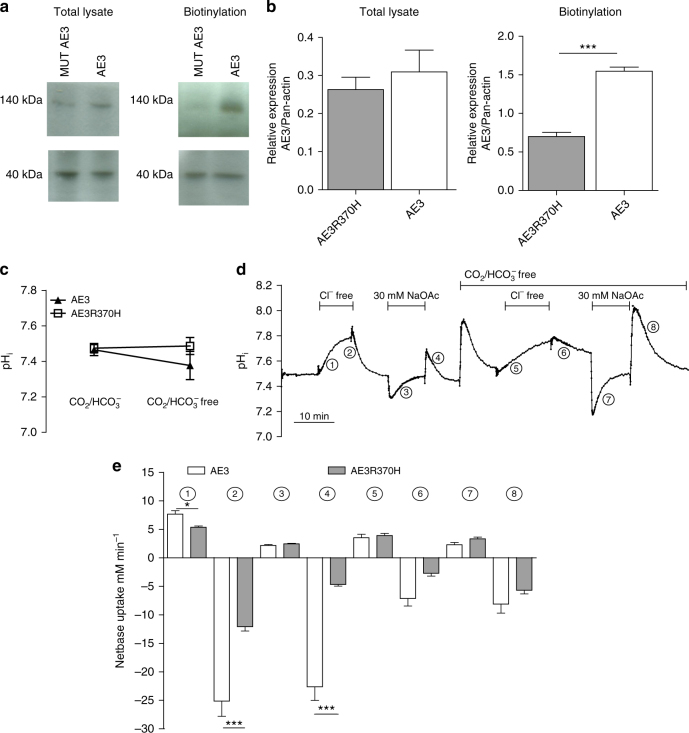
Immunoblot of AE3 and intracellular pH in HEK-293 cells. **a** Representative immunoblots of total and membrane localized (biotinylated) AE3 protein in AE3 transfected and AE3_R370H_ transfected HEK-293 cells. Pan-actin was used as control/reference protein. **b** Average total and membrane localized AE3 protein relative to pan-actin expression (*n* = 4) (*p < 0.05 as determined by unpaired Student t-test). **c** Steady state pH_i_ of AE3 transfected and AE3_R370H_ transfected HEK-293 cells in CO_2_/HCO_3_^−^ containing media and CO_2_/HCO_3_^−^ free media. **d** Representative recording of pH in HEK-293 cells. Numbers indicate parts of the protocols used for calculations of net base transport shown in **e**. **e** Average net base uptake for AE3 transfected and AE3_R370H_ transfected HEK-293 cells. Numbers correspond to the numbers given in **c** (*n* = 7). *p < 0.05, **p < 0.01, ***p < 0.001 as determined by unpaired Student t-test. Error bars throughout represent the s.e.m.

We functionally characterized the mutated AE3 variant in HEK-293 cells transfected with cDNA encoding wild-type AE3 or the AE3-R370H mutant. No difference in expression of wild-type and mutated AE3 was seen in whole cell lysates (Fig. 2a and Supplementary Fig. 1), however, the surface localization of the mutated protein was significantly reduced compared to the wild-type protein (Fig. 2b). This strongly suggests that trafficking of the mutated AE3 to the membrane was compromised.

### Reduced Cl,HCO_3_-exchange activity of mutated AE3

Steady state pH_i_ was similar in HEK cells expressing wild-type AE3 and mutated AE3 in both the presence and absence of HCO_3_^−^ (Fig. 2c). We next analyzed HCO_3_^−^ transport in the two types of cells (Fig. 2d, e). When assayed in the presence of HCO_3_^−^, the net transfer rate of acid equivalents across the cell membrane following both omission of Cl^−^ and addition of Cl^−^ was faster in the cells expressing wild-type AE3 compared to cells expressing the mutated variant. In the absence of HCO_3_^−^, the transfer rates were low and no difference was seen (Fig. 2d, e). These findings indicate a loss-of-function effect of the mutation. Addition of sodium acetate (NaOAc) was associated with a reduction of pH_i_ followed by recovery. When NaOAc was washed out, pH_i_ increased similarly in both groups. However, the following decrease in pH_i_ (in the presence of HCO_3_^−^) was significantly faster in cells expressing wild-type AE3 compared to cells expressing the mutated variant (Fig. 2d, e), further supporting a loss-of-function effect.

### Elevated pH_i_ and short QTc in AE3−deficient zebrafish hearts

**Fig. 3:**
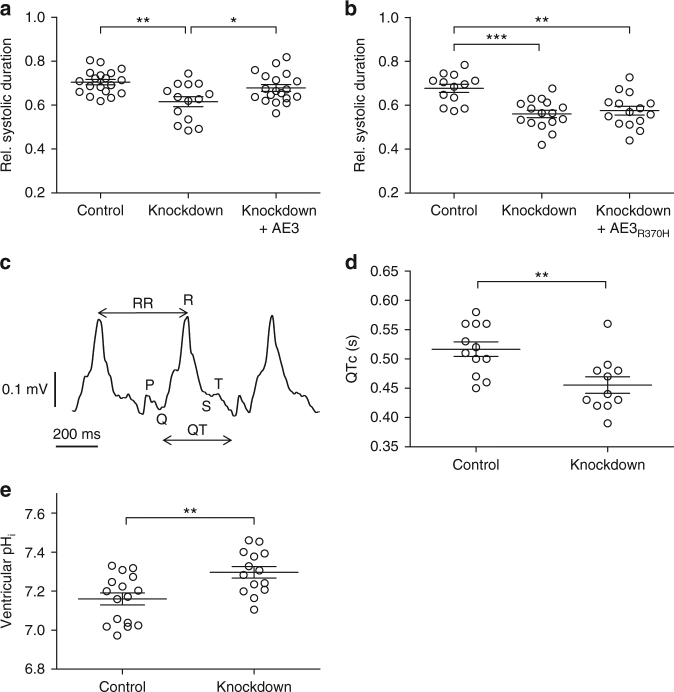
Loss of *slc4a3* in zebrafish embryos replicates the QTc shortening observed in patients. **a** Systolic interval divided by heart cycle duration is significantly reduced in *slc4a3* knockdown embryos 2 days post fertilization (dpf). This phenotype is rescued by overexpression of AE3 in knockdown embryos. *n* = 19 (Control), *n* = 14 (Knockdown), and *n* = 19 (Knockdown + AE3) compiled from three independent experiments, each including all three experimental groups. **b** Reduced systolic interval in *slc4a3* knockdown embryos is not rescued by overexpression of R370H-mutated AE3 in knockdown embryos. *n* = 13 (Control), *n* = 16 (Knockdown), and *n* = 15 (Knockdown + AE3_R370H_) compiled from three independent experiments, each including all three experimental groups. **c** A raw ECG recording of a 2 dpf zebrafish embryo; P,Q,R,S,T waves, and RR and QT intervals indicated. **d** Corrected QT intervals (QTc) for control and *slc4a3* knockdown embryos were calculated with Bazett’s formula. *n* = 12 (Control) and *n* = 11 (Knockdown) compiled from three independent experiments, each including both experimental groups. **e** pH_i_ is significantly increased in hearts from *slc4a3* knockdown embryos 2 days post fertilization (dpf). *n* = 16 (Control), *n* = 14 (Knockdown). ^*^ *p* < 0.05, **p < 0.01, ***p < 0.001 as determined by one way ANOVA with Tukey’s post-test (**a**, **b**) or unpaired student t-test (**d**, **e**). Error bars throughout represent the s.e.m.

To determine the consequence of lacking AE3 activity in vivo, we assessed the effect of *slc4a3* knockdown on systolic contraction in zebrafish. Zebrafish *slc4a3* was efficiently knocked down in embryos (Supplementary Fig. 2a). Whereas heart rates were unaffected by the knockdown (Supplementary Fig. 2b and Supplementary Fig. 2c), systolic contraction duration was significantly shortened (Fig. 3a). The phenotype was rescued by mRNA encoding wild-type, but not Arg370His-mutated AE3, demonstrating that amino acid residue Arg370 of AE3 is required to maintain normal systolic duration in vivo (Fig. 3a, b). Importantly, ECG analysis (Fig. 3c) demonstrated that the observed shortening of systolic contraction corresponded to significantly shortened QTc for *slc4a3* knockdown embryos (Fig. 3d) which was associated with an increased ventricular pH_i_ (Fig. 3e).

### High pH_i_ and low [Cl^−^]_i_ shorten cardiac action potential

**Fig. 4:**
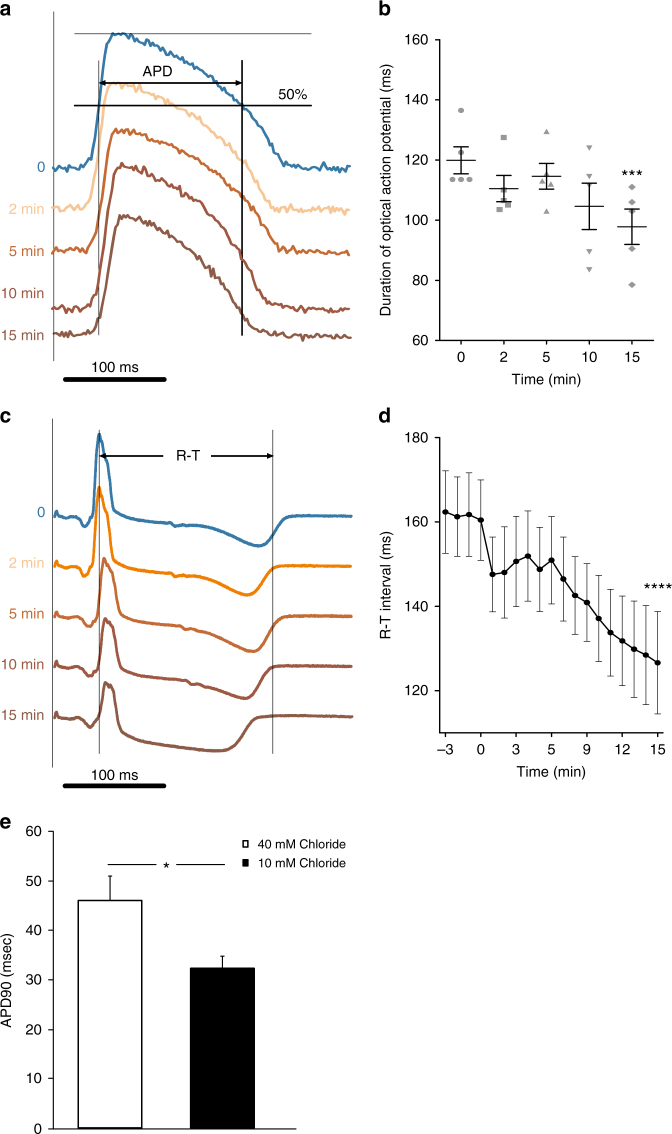
Intracellular alkalization and low [Cl^−^]_i_ shortens the action potential duration and R-T interval. **a** Action potentials of rabbit hearts measured before and during application of 10 mM NH_4_Cl. APD: Action potential duration. **b** Mean APD during application of NH_4_Cl (*n* = 5). Zero indicates time of intervention. **c** ECGs in rabbit hearts before and during application of 10 mM NH_4_Cl. **d** Mean RT interval in rabbit hearts (*n* = 5). In **a**, **c** the records are aligned at the pacing stimulus. ***p < 0.001, ****p < 0.0001 as determined by one-way repeated measures ANOVA. Zero indicates time of intervention. **e** Bar graph showing APD90 of rat ventricular cardiomyocytes patched with 40 mM (open, *n* = 23) or 10 mM (black, *n* = 16) chloride in the pipette solution. *p < 0.05 as determined by one-way repeated measures ANOVA. Error bars throughout represent the s.e.m.

Due to the increased pH_i_ observed in embryonic *slc4a3* knockdown zebrafish hearts, we tested whether increased pH_i_ leads to shortened APD and QTc in the isolated heart. Addition of 10 mM NH_4_Cl extracellularly caused an increase of pH_i_ in rabbit cardiomyocytes as expected (Supplementary Fig. 3). The increased pH_i_ was associated with a reduced APD of the cardiomyocytes (Fig. 4a, b) and a shorter RT (Fig. 4c, d). Since AE3 mediates HCO_3_^−^ efflux in exchange for influx of Cl^−^, we further tested whether lowering of [Cl^−^]_i_ shortens APD by patching rat ventricular cardiomyocytes with 40 mM or 10 mM Cl^−^ in the pipette solution. As shown in Fig. 4e, 10 mM Cl^−^ was associated with a shorter APD compared to 40 mM Cl^−^.

## Discussion

We report that a mutation in the *SLC4A3* gene, which encodes the cardiac chloride-bicarbonate exchanger AE3, is associated with SQTS. Within a large affected family, the group of carriers had experienced several serious cardiac events and SCD, whereas no events had occurred in non-carriers. All carriers had a QTc ≤ 370 ms and all non-carriers had a QTc > 370 ms. The J-point to T-peak interval was ≤ 140 ms in all carriers. Jp-Tp has been suggested as a parameter to be applied in the diagnostic score of SQTS^4^, and has been reported to have a high specificity for the SQTS and is clinically readily recognizable on visual ECG inspection. These ECG findings indicate a high penetrance as assessed by repolarization duration, the equivalent of the APD. In agreement with findings in other SQTS families, clinical manifestations were present in males only, and absent in children. Out of 27 living mutation carriers from the two families, six showed cardiac events considered SQTS related. The mutation leads to reduced trafficking of AE3 to the membrane, loss of Cl,HCO_3_-exchange and increased pH_i_. The role of the mutation was emphasized by the identification of the same mutation in an unrelated family with SQTS and by the finding that *slc4a3* knockdown in zebrafish causes increased pH_i_, short QTc, and reduced systolic contraction duration, which was rescued by wildtype but not R370H-mutated AE3. Thus, our data show the involvement of an anion transporter in the development of SQTS.

Previously, other *SLC4A3* variants have been associated with seizures^17^. Further, mice with a targeted disruption of the Cl,HCO_3_-exchanger AE3 display a reduced threshold for triggering an epileptic seizure^18,19^. However, none of the mutation-carriers in our two families has had any clinical presentations with unconsciousness suspected to be due to epileptic events. The case with aSCD had documented VF.

AE3 is a comprehensively studied Cl,HCO_3_-exchanger with the highest mRNA expression of the HCO_3_^−^ transporters^20^ in mouse hearts. AE3 is known to be important for myocardial pH_i_ recovery from alkaline loads^20,21^. However, AE3 knockout mice show no overt cardiac phenotype unless crossed with Na,K,Cl^−^exchanger knockout mice^22^ or mice carrying a mutation in α-tropomyosin in a model of hypertrophic cardiomyopathy^23^. No electrophysiological characterization of the hearts of the AE3 knockout mice has been published though.

**Fig. 5:**
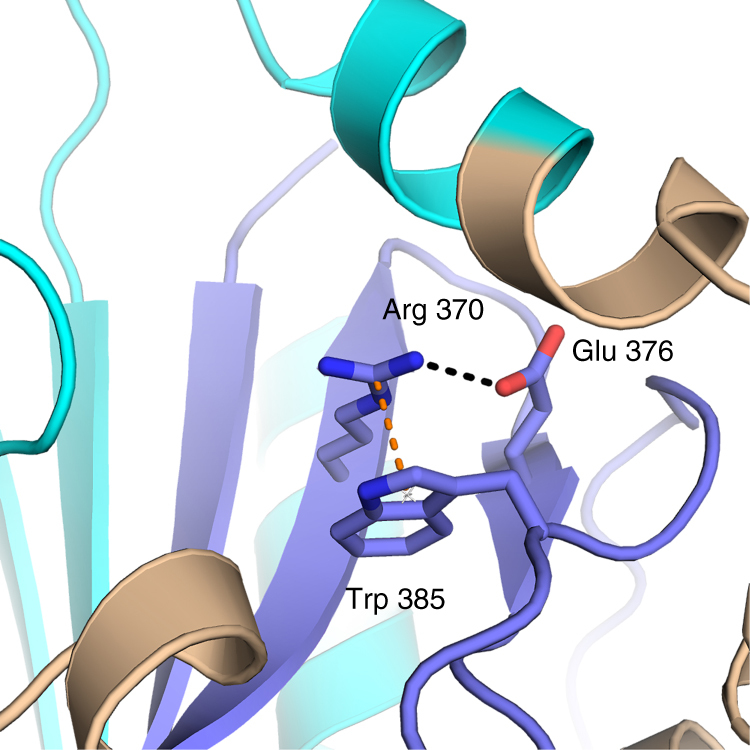
Molecular interaction in direct contact with Arg370. Glu376 is forming a charged hydrogen bond to Arg370 (dashed black lines), while Trp385 is linked through a cation-π interaction (orange dashed line). The figure was prepared with The PyMOL Molecular Graphics System, Version 1.8 Schrödinger, LLC, and the modeling based on an alignment of the AE1 and AE3 sequence in the cytoplasmic region using PDB ID entry 1HYN as template

The identified mutation of the *SLC4A3* gene causes a single-residue Arg-to-His change at amino acid position 370 of AE3, which is part of the E_367_-T-A-R-W-I-K-F-E-E_376_ motif. This motif is highly conserved in all SLC4 gene products^24^. Previously, a Glu-to-Arg mutation in position 91 of NBCe1/SLC4A4 (position equivalent to Glu375 of AE3)^25^ has been found to cause poor surface targeting upon expression in MDCK cells. By analysis of an NBCe1 homology model, Arg370 has been proposed to interact with Glu376 (equivalent to Glu92 in NBCe1) through a salt bridge^26^. We find by further homology modeling that in addition, Arg370 may form a cation-π interaction with Trp385 (Fig. 5). These two structural elements hint to a fixed structural environment that spatially orientates Arg370 and could lead to misfolding if disrupted with a mutation to histidine. In agreement with these data, we found that the mutated variant (Arg-370-His) showed a level of translation similar to wild-type AE3, but only poor surface targeting.

The reduced membrane trafficking is strongly supported by the reduced Cl,HCO_3_-exchange activity observed in transfected cells expressing the mutated variant. The data therefore strongly suggest that the mutation leads to trans-membraneous Cl^−^ and HCO_3_^−^ transport deficits, which might increase pH_i_ and reduce [Cl^−^]_i_.

There is some evidence that modulation of AE3 activity leads to changes in pH_i_ and [Cl^−^]_i_. In AE3 KO mice there is a tendency to a higher steady state pH_i_ in isolated cardiomyocytes compared to pH_i_ in cardiomyocytes from WT mice and the recovery time from an imposed intracellular alkalosis is significantly prolonged^27^. This is consistent with the effect of an inhibitory AE3 antibody on pH_i_ in rat cardiomyocytes. Thus, in the presence of endothelin-1, inhibition of AE3 increased pH_i_^21^. Additionally, α-adrenergic receptor activation of the rat heart reduces steady state pH_i_ most likely through activation of AE3^28^. These findings indicate that in hearts expressing the mutated AE3, the capacity to counteract an increase in pH_i_ is compromised, particularly under in vivo conditions where the heart is exposed to various hormones and neurotransmitters. Our finding of an increased steady state pH_i_ in the hearts of zebrafish embryos with knockdown of AE3 is consistent with this. It has been suggested that activation of AE3 during ischemia^29^ and extracellular acidosis^30^ contribute to the increase in guinea pig and sheep ventricular muscle [Cl^−^]_i_. This indicates that the effect of AE3 on [Cl^−^]_i_ may also depend on conditions. Although these findings suggest that inhibition of AE3 mediated anion transport leads to increased pH_i_ and decreased [Cl^−^]_i_ this conclusion may need further validation.

Since the AE3 mutation increased pH_i_ and likely decreases [Cl^−^]_i_, we tested the effects of increased pH_i_ and decreased [Cl^−^]_i_ on myocyte electrophysiology. Increased pH_i_ was associated with a shortened APD in rabbit heart. This finding is consistent with the finding of an APD prolongation by low pH_i_ in rabbit ventricular myocytes^12^. It is unclear how this effect is mediated. However, high pH_i_ activates Kv7.1 channels (KCNQ1)^31,32^, which may accelerate repolarization. The knowledge about the effects of high pH_i_ on cardiac ion channels seems very limited. For *KCNH2* we are not aware of studies addressing the effect of high pH_i_. For the L-type Ca^2+^ channels the effect of high pH_i_ is likely inhibition^12^ which would also accelerate repolarization.

We demonstrated that also reduced [Cl^−^]_i_ leads to shortened APD. A reduction of [Cl^−^]_i_ will cause the equilibrium potential for Cl^−^ to be more negative and hence increase the Cl^−^ influx in phase 2 and the beginning of phase 3 leading to a shorter action potential. This finding is consistent with studies where different Cl^−^ channels in cardiomyocytes are inhibited^33^. Together this demonstrates that low [Cl^−^]_i_ due to inhibition of AE3 activity might contribute directly to the SQTS.

We suggest that both high pH_i_ and low [Cl^−^]_i_ are likely to contribute to the SQTS, but cannot exclude that other effects may also contribute.

The low event rate during follow-up indicates the difficulties in predicting the risk of ventricular arrhythmias or SCD in patients with SQTS and underscores the importance of developing risk scores. The clinical course of more families with AE3 mutations—probably to be identified in the pool of SQTS patients with hitherto negative genetic test results—will be needed to identify AE3 mutation specific clinical risk factors.

In summary, we have identified a novel mutation in AE3 in two independent families with SQTS. The mutation leads to defective targeting of AE3 to the cell membrane, reduction of Cl,HCO_3_-exchange and increased pH_i_. The impact of the mutation on QTc duration was confirmed in zebrafish and the mechanism through which a defect in Cl,HCO_3_-exchange leads to shortened QTc likely relates to a combined effect of a decrease in HCO_3_^−^ efflux (i.e., increased pH_i_) and a decrease in Cl^−^ influx. These findings offer insight into mechanisms of arrhythmia in general and may provide new treatment possibilities in patients with SQTS. Currently, no specific factors triggering arrhythmia in patients with SQTS have been reported. Based on this study, possible triggers may include medications, physical activities, metabolic derangements or diets challenging the Cl,HCO_3_-exchange.

Further studies are required to determine the frequency of involvement of *SLC4A3* in SQTS and to delineate details of how high pH_i_ and low [Cl^−^]_i_ may lead to the serious arrhythmia associated with SQTS.

## Methods

### Patients and clinical evaluation

Informed consent was obtained from all subjects. The study was approved by the Ethics Committee Central Denmark Region (no. 1-10-72-189-16) and by the Danish Data Protection Agency (record no. 1-16-02-629-16).

A 31-year old man resuscitated after cardiac arrest was diagnosed with SQTS^4^. Cascade screening revealed a large family with 41 relatives to the proband. Subsequently, another unrelated family with SQTS was included for verification. Clinical work-up included clinical examination, ECG, echocardiography, routine blood tests and 48-hours Holter monitoring. Bazett corrected QT interval (QTc) was used. Blood was collected from all family members for molecular genetic analysis. In two cases, post-mortem genetic testing was performed.

### Exome sequencing and data analysis

Genomic DNA was extracted from peripheral leucocytes or paraffin tissue blocks on the QiaSymphony using standard protocols (Qiagen). DNA from the index patient was initially analyzed with the MOMA NGS Heart panel v1 (Custom EZ in solution DNA Capture, Nimblegen), see Supplementary Table 2 for gene list using MiSeq sequencing (Illumina). For whole exome sequencing 1 µg DNA was used for library preparation using the TruSeq DNA LT Sample Prep Kit v2 according to the manufacturer’s instructions (Illumina) followed by hybridization using Nimblegen SeqCap EZ Exome v3 (Roche) and Paired-end Sequencing (2 × 100 bp) on the Illumina HiSeq 2000 with TruSeq v3 chemistry (Illumina). CLC BioMedical Genomics Workbench 2.1.1 (Qiagen) was used for data analysis and data was trimmed for adaptor sequence and ambiguous bases before alignment to GRCh37, duplicate removal and SNV and indel calling with the fixed ploidy variant caller. Variants were uploaded to Ingenuity Variant Analysis tool (Qiagen) and filtered to exclude variants with an allele frequency of at least 5% in either 1000 Genomes Project^34^, ExAC^35^ or NHLBI ESP exomes (http://evs.gs.washington.edu/EVS). Finally, the variants shared by all individuals with SQTS but not observed in any of the control samples were subjected to further analysis. PCR amplification and subsequent Sanger sequencing of exon 8 containing the variant was used in all included family members for cascade screening for the *SLC4A3* variant.

### Construction of AE3 R370H and gene expression

Human *SLC4A3* (NM_201574) cDNA clone (Origen; Clone SC308062, US) was used as template for introducing the mutation. The point mutation, was introduced using a phusion site-directed mutagenesis kit (Thermo Scientific) and amplified with modified DNA oligonucleotides (Europhins MWG) holding the mutation. Plasmid DNA carrying either AE3 or AE3_R370H_ (controlled for amplication errors) was transfected into HEK-293 cells (ATCC, catalogue number CRL-1573) using TransIT-LT1 transfection reagent (Mirus). Cells were shifted to selection media containing 600 µg ml^−1^ G418 (Sigma Aldrich) 48 h posttransfection. Cells for pH_i_ experiment were grown in poly-Lysine coated dishes.

### AE3 surface processing and western blot analysis

Surface proteins were isolated from HEK-293 cells using the biotinylation based Pierce cell surface protein isolation kit (Thermo Scientific). Total protein cell lysate was prepared by resuspending and homogenizing cells in lysis buffer (10 mM Tris–HCl, 250 mM sucrose, 1 mM EDTA, 1 mM EGTA, 2% Triton X-100) containing Complete Mini, EDTA-free protease inhibitor (Roche Applied Science, Germany, 1 tablet per 10 ml solution). Protein concentration was assayed (Pierce BCA Protein Assay kit; Thermo Scientific) and 10 µg of protein were solubilized in SDS-buffer and separated by SDS-page. Proteins were transferred to PVDF membranes (Amersham;GE Healthcare). Membranes were incubated with primary antibodies (Rabbit anti-AE3 (1:2000), kindly provided by Professor S. Alper, (Renal Division and Division of Molecular and Vascular Medicine, Beth Israel Deaconess Medical Center, Boston, USA), Rabbit-anti-pan-actin (1:2000), Cell Signaling Technology Inc., USA [Catalog no. 4968]) overnight. Secondary antibody (goat-anti rabbit; Millipore, US (1:2000)) linked to horseradish peroxidase were used to detect binding with the ECL chemiluminescence system (Amersham; GE Healthcare).

### pH_i_ measurements in HEK-293 cells

Cells were loaded with 2 µM of the pH sensitive dye BCECF-AM for 15 min in a physiological salt solution (PSS) at 37 °C. The cells were placed on an Olympus IX70 microscope with an Olympus LUCPlanFL N 20× objective (N.A. 0.45) and an EasyRatioPro fluorescence imaging system (Photon Technology International). The cells were excited alternately at 485 and 440 nm and emission collected at 550 nm. The BCECF ratio between emission light collected during excitation at 485 and 440 nm, respectively, was calculated after subtraction of background fluorescence and calibrated to pH units using the high-K^+^ nigericin method, as previously described^36^. PSS contained in mM: 139 Na^+^, 5.9 K^+^, 1.6 Ca^2+^, 1.2 Mg^2+^, 122 Cl^−^, 25 HCO_3_^−^, 1.2 SO_4_^2−^, 1.2 H_2_PO_4_^−^, 10 HEPES, 5.5 glucose and 0.026 EDTA. In HCO_3_^−^ free solutions HCO_3_^−^ was replaced by Cl^−^. In Cl^−^ free solution Cl^−^ was substituted with aspartate. HCO_3_^−^ containing solutions were gassed with 5% CO_2_ in air; HCO_3_^−^ free solutions were gassed with air. All solutions were titrated to pH 7.4. After washout of BCECF-AM steady state pH_i_ was recorded and the solutions modified, as shown in Fig. 2d. Intracellular buffering capacity was calculated from the change in pH_i_ following washing and washout of NaAcetate (NAOAc). The transport rate of acid equivalents across the membrane was calculated from the change in pH_i_ per unit time multiplied by the buffering capacity.

### Animal ethics

All animal experimental procedures were conformed to guidelines from the European Convention for the Protection of Vertebrate Animals used for Experimental and other Scientific Purposes and were approved by and conducted with permission from the Animal Experiments Inspectorate of the Danish Ministry of Environment and Food, and the State Food and Veterinary Service of the Republic of Lithuania and Ethics Committee of the Lithuanian University of Health Sciences.

### Zebrafish knockdown of *slc4a3* and phenotyping

Adult zebrafish of the AB wildtype strain were fed four times daily and maintained on recirculating housing systems on a 14 h light per 10 h darkness cycle at 28 °C. Embryos were obtained by natural crosses and maintained in E3 medium (5 mM NaCl, 0.17 mM KCl, 0.33 mM MgSO_4_, 10^–5^% (w/w)methylene blue, 0.33 mM CaCl2, 2 mM Hepes pH 7.4) at 28 °C. Knockdown was performed by microinjection into zygotes of *slc4a3*-targeted morpholino e7i7 (TCAGAAGGCTCAGCTCTCACCTCAT) or standard control morpholino (CCTCTTACCTCAGTTACAATTTATA, Gene Tools, LLC). e7i7 efficiency was assessed by reverse transcription PCR from cDNA synthesized from total RNA extracted from groups of ten microinjected embryos two days post fertilization (2 dpf) using primers 5′-CTTCGAGGACAATCCTGGGG-3′/5′-CTACGGAAGGACAGTGAGGC-3′ (*slc4a3*) and 5′-CACGAGACCACCTTCAACT-3′/5′-ATCCAGACGGAGTATTTGC-3′ (*β-actin*). Human *SLC4A3* mRNA was in vitro synthesized using the mMessage mMachine T7 ULTRA kit (Ambion) with linearized wild type (NM_201574) or R370H-mutated AE3-encoding plasmid as template. Similar stability of microinjected wild type and mutant mRNA was verified by RT-PCR on 2 dpf zebrafish embryos using the human AE3-specific primer set 5′-ATCATCACCCAACTCCCAGC-3′/5′- GGCACTTAGGAGGTCTTGCC-3′.

For pH_i_ measurements in isolated zebrafish hearts, 2 dpf zebrafish embryos were briefly anesthetized by rapid cooling on wet ice prior to the dissection of the heart from the embryo. The isolated heart was immediately transferred to fish buffer and the embryo was euthanized. The hearts were loaded with 5 µM BCECF-AM for 5–15 min in fish buffer at room temperature. After washout of BCECF-AM the recording and calibration of the fluorescence signal from the ventricles of the hearts were made as described for pH_i_ measurements in HEK cells. The fish buffer contained in mM: 131 Na^+^, 4.0 K^+^, 1.6 Ca^2+^, 127 Cl^−^, 10 HCO_3_^−^, 1.0 H_2_PO_4_^−^, and 5.0 glucose. The solution was gassed with 1% CO_2_ in air and pH was 7.65.

For assessment of cardiac function, 2 dpf embryos were anaesthetized using buffered 150 ng mL^−1^ Ethyl 3-aminobenzoate methanesulfonate (MS-222, Sigma-Aldrich) and staged in 4% methyl cellulose (average *M*_n_ ~ 86,000, Sigma-Aldrich) in E3. To obtain systolic and diastolic durations, high-speed video recordings of eight seconds duration at 100 frames per second were made. Video analysis was performed, as previously described^37,38^ using an algorithm written in MATLAB (The MathWorks Inc.) allowing for evaluation of ventricular contraction and relaxation by quantification of changing pixel intensity. Recordings were excluded from analysis in cases where (1) atrial contraction/relaxation caused movement of the ventricle interfering with quantification of ventricular contraction/relaxation and (2) the quality of recording, e.g., signal-to-noise ratio, did not allow quantitative analysis. All embryos were euthanized by MS-222 overdose immediately after phenotyping.

For ECG recordings two dpf zebrafish embryos were placed in E3 medium with MS-222 (150 ng mL^−1^) for ECG at room temperature (22–24 °C). The recordings were made using a potassium acetate (3 M) filled borosilicate glass micropipette (PG15OT-7.5; Harvard Apparatus) with tip resistances 5–10 MΩ at 10 kHz using an Axopatch 200B amplifier (Axon Instruments, Inc.) in a current-clamp configuration. The recording electrode was positioned on embryo skin surface near the heart (no penetration). Data acquisition was done with the software package Clampex 7 for Windows (Axon Instruments Inc.). Data were analyzed in LabChart 7 (ADInstruments) after low-pass digital filtering with cut-off frequency 50 Hz. The ECG data were only excluded if the quality of recording, i.e., signal-to-noise ratio, did not allow quantitative analyzes of QT intervals. QT and RR intervals were calculated for each embryo and converted to QTc.

### Measurement of action potentials and ECG in rabbit heart

Experiments were performed on six months old male New Zealand white rabbits (weight: 2.95 ± 0.18 kg, *n* = 5). Animals received 10 mg kg^−1^ xylazine (s.c.) following i.v. injection of ketamine (10 mg kg^−1^) with heparin (1000 U kg^−1^). The heart was excised and coronary arteries perfused in the Langendorff-system with oxygenated Tyrode solution (in mM): 135 NaCl, 5.4 KCl, 1.8 CaCl_2_, 0.9 MgCl_2_, 0.33 NaH_2_PO_4_, 10 glucose, 10 HEPES, pH 7.4, 37 °C. In total 20 μM (−)-blebbistatin and 5 mM 2,3-butanedione monoxime were added to stop contraction. The heart was stimulated with bipolar silver electrodes in the left ventricle (LV) at 300 ms interval and 2 ms square pulses of about twice diastolic threshold. ECG was recorded with two ECG electrodes placed on LV (near the apex and near the base) using FE136 amplifier (ADInstruments) and digitized at 20 kHz.

The heart was stained with 50 μM di-4-ANBDQBS (JPW-6033) by slow 10 ml bolus injection. Optical recordings of action potentials (OAP), as described previously^39^ were acquired with a cooled (−100 °C) 14-bit-EMCCD camera (iXonEM + DU-860, Andor Technology) with a 50-mm focal length objective (Navitar) and using the imaging software (Andor SOLIS). Collimated LED of 660 nm wavelength (M660L3, Thorlabs) was used for excitation of di-4-ANBDQBS. Fluorescence emission was collected using long-pass filter of 720 nm (HQ720lp, Chroma) with a sampling rate of 500 Hz. Background fluorescence was subtracted and 16 consequent recordings were averaged and normalized to the control level. No exclusion criteria were applied to the data from the rabbit hearts. Di-4-ANBDQBS was obtained from Dr L. Loew (University of Connecticut, USA).

LabChart8 Pro software (ADInstruments) was used to analyze the R-T interval between R wave and 50% of T wave amplitude decrease. The optical movies were preprocessed with the use of ImageJ software and OAPs were analyzed using MS Excel Activation time of OAP was calculated as time between stimulus and 50% of OAP upstroke amplitude; duration of OAP (OAPD)—time between 50% of OAP upstroke amplitude and 50% of OAP repolarization time.

### Recording of action potentials and ECG in rat cardiomyocytes

Ventricular cardiomyocytes were isolated from male Sprague-Dawley rats (weight: 325 ± 8.3 g, *n* = 13) (Taconic, Ll. Skensved, Denmark). Rats were anaesthetized using isoflurane (5% in 100% O_2_), hearts were quickly excised and the aorta cannulated. Hearts were retrogradely perfused by a solution containing in mM: 136 NaCl, 2.68 KCl, 1.47 KH_2_PO_4_, 8.06 Na_2_HPO_4_, 0.9 CaCl_2_, 0.9 MgCl_2_, 6 glucose, pH 7.4, equilibrated with 100% O_2_ for 5 minutes, followed by 2 minutes of perfusion with the same solution without calcium. Subsequently, perfusion was changed to a solution containing (in mM) 20 NaCl, 120 K-gluconate, 1 MgCl_2_, 10 HEPES, 10 glucose, pH 7.4 equilibrated with 100% O_2_ for 2 min and perfusion with the same buffer added 96 U ml^−1^ collagenase (Type IV, Worthington), 25 µM CaCl_2_ and 0.1% BSA until the tissue was digested. After the perfusion process, the heart was transferred to a petri dish, the atria were discarded and the ventricles were cut into pieces. A solution containing (in mM) 100 K-glutamate, 10 K-aspartate, 25 KCl, 10 KH_2_PO_4_, 2 MgSO_4_, 20 Taurine, 5 Creatine, 20 glucose, 0.5 EGTA, 5 HEPES, 0.1% BSA, pH 7.4 was used to cleanse the remaining pieces from collagenase and to stop the digestive process. The pieces of ventricle were gently bubbled with 100% O_2_ to dissolve the tissue and release the cardiomyocytes. Hereafter the solution was filtered using a metal grid (50 mesh) and washed. Ca^2+^ was gradually reintroduced to a final concentration of 0.72 mM and the cells were stored at 5 °C, until use on the day of isolation. Action potentials were recorded in the whole-cell zero current patch clamp configuration using a SEC-05LX amplifier (npi electronics, Tamm, Germany). Signals were low-pass filtered (current: 2 kHz; potential: 1 kHz) and sampled at 10 kHz using CellWorks (npi electronics, Tamm, Germany).

The cardiomyocytes were superfused at room temperature with solution containing in mM: 136 NaCl, 2.68 KCl, 1.47 KH_2_PO_4_, 8.06 Na_2_HPO_4_, 0.9 CaCl_2_, 0.9 MgCl_2_, 6 glucose; pH adjusted to 7.4. Borosilicate patch pipettes (3–5 MΩ) were filled with either 40 or 10 mM Chloride containing solution. The 40 mM Chloride solution contained (in mM): 105 K-gluconate, 40 KCl, 9 Na-gluconate, 5 Na-pyruvate, 1 Na_2_ATP, 1.92 MgSO_4_, 0.353 Ca-gluconate_2_, 1 EGTA, 5 HEPES, pH adjusted to 7.2. For 10 mM Chloride, 30 mM KCl was exchanged with K-gluconate. Action potentials were elicited at 1 Hz by current injection through the pipette for ten minutes. The reported APD90 value is the average time to 90% repolarization of the last 20 stimuli. Analyses were performed using a custom written script in MatLab (MatLab R2013a, MathWorks, Inc.). Data from the patch clamp analysis were excluded if the resting potential was unstable, in such cases depolarization most often led to hypercontracture and loss of seal stability. These are standard criteria for patch clamp experiments in our laboratory.

### Statistics

Clinical data are presented as mean values ± SD. Statistical significant differences between patient groups were ascertained by Students two-tailed *t*-test for un-paired observations. Bonferroni’s correction was applied to correct for multiple comparisons. Experimental data are presented as mean values ± s.e.m. Differences between means in the HEK cell and rat cardiomyocyte experiments were tested with unpaired Student t-test. Differences between means in the zebrafish embryos were tested by unpaired Student t-test or one-way ANOVA with Tukey’s post-test. Differences between means in the rabbit heart intracellular alkalization experiments were tested with one-way repeated measures ANOVA. *p* < 0.05 was considered significant. All clinical and zebrafish embryo data were tested and found to be normally distributed. The pH measurements and the action potential measurements are known from other experiments to have a normal distribution.

Sample sizes were estimated based on previously published data for the experiments on zebrafish embryos, rabbit hearts, and rat cardiomyocytes. No statistical method was used to determine sample size. No randomization or blinding was applied to experiments involving zebrafish embryos, rabbit hearts, and rat cardiomyocytes.

### Data availability

Exome sequencing data have been deposited in The European Genome-phenome Archive under accession code EGAS00001002632. Access to the sequence data needs to be approved by the local Data Access Committee.

## Electronic supplementary material

Supplementary Information
