# Supplementary material for: Loss-of-activity-mutation in the cardiac chloride-bicarbonate exchanger AE3 causes short QT syndrome
Source: Nat Commun. 2017 Nov 22;8:1696. doi: 10.1038/s41467-017-01630-0 (PMC5700076; doi:10.1038/s41467-017-01630-0)
Supplement: Supplementary file 1 — Supplementary Information [file 41467_2017_1630_MOESM1_ESM.pdf]

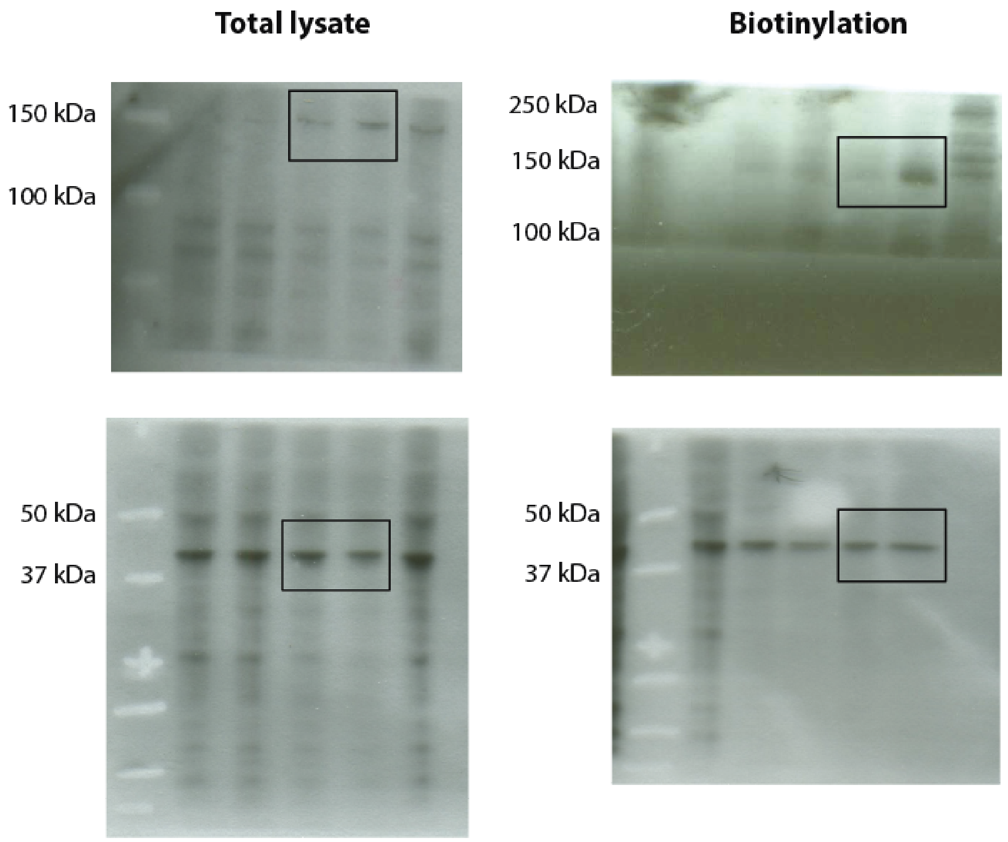

Supplementary Figure 1. Full-length western blot images. Uncropped full-length pictures of Western blotting membranes. Boxes indicates cropped blot image in the figure 2a.

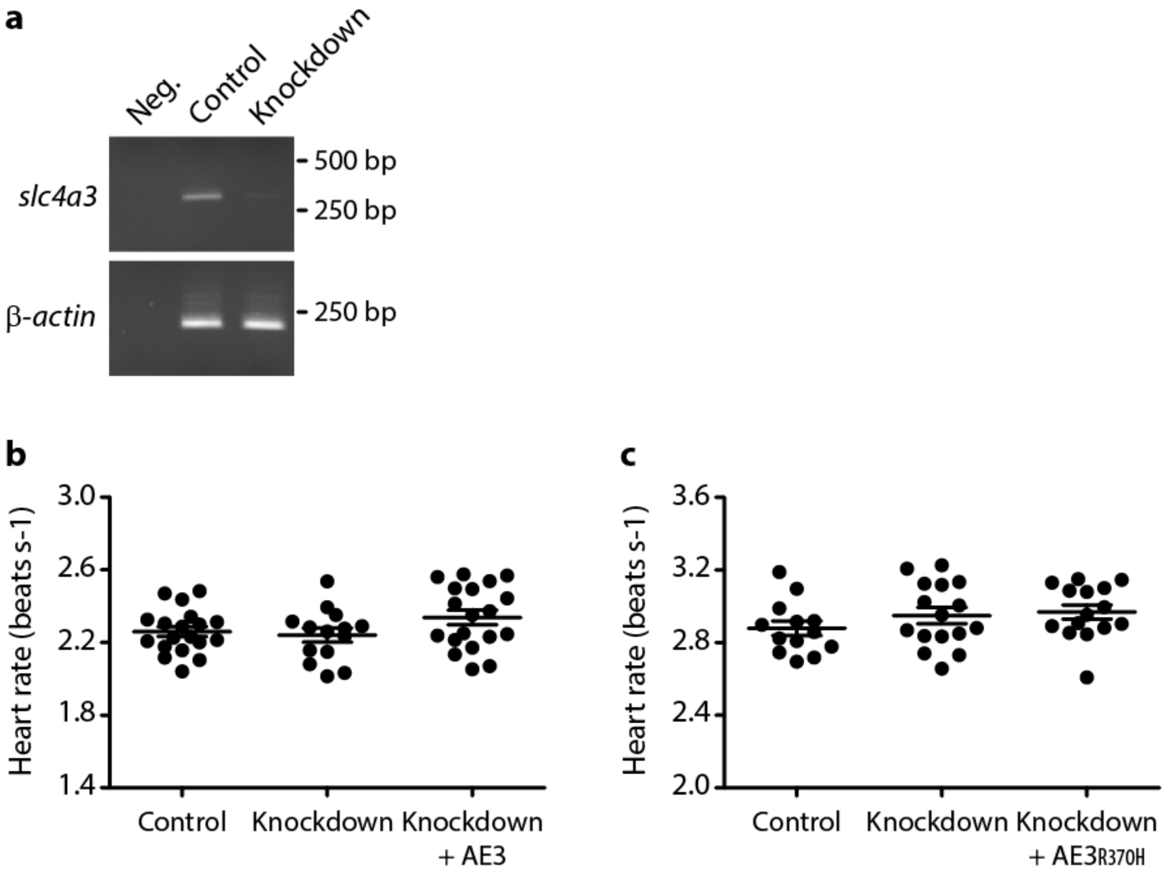

Supplementary Figure 2. Efficient *slc4a3* knockdown does not affect heart rate.

a. *slc4a3* is efficiently knocked down by microinjection of *slc4a3*-targeted splice-inhibiting morpholino e7i1 as assessed by RT-PCR. Neg: no-template PCR control, Control: 7 ng standard control morpholino, Knockdown: 7 ng e7i7. β-*actin* PCR is used as loading control. Each sample is a pool of 10 embryos. **b,c** Heart rate 2 dpf is not affected by *slc4a3* knockdown, combined *slc4a3* knockdown and AE3 overexpression (b), or combined *slc4a3* knockdown and $AE3_{R370H}$ overexpression (c). n=19 (Control), n=14 (Knockdown), and n=19 (Knockdown + AE3) compiled from three independent experiments, each including all three experimental groups (b), and n=13 (Control), n=16 (Knockdown), and n=15 (Knockdown + $AE3_{R370H}$) compiled from three independent experiments, each including all three experimental groups (c). Error bars throughout represent the s.e.m.

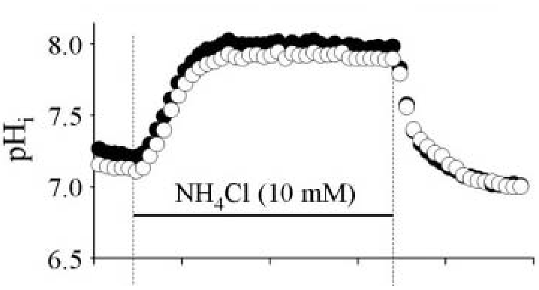

Supplementary Figure 3. Effect of NH_4Cl on intracellular pH. Intracellular pH was monitored in two cardiomyocytes after loading with 4 µM of the pH-sensitive fluorescent dye BCECF via patch pipettes.

Supplementary Table 1. Clinical features of all family members.

Family/ relatives	Gender	Clinical presentation / age	QT (ms)	QTc (ms)	Jp-Tp ms	SLC4A3 c.1109 G>A	ICD implantation
1 (I:1)	Male	Dead / 85 y (father SCD young)	na	na	na	na	na
1 (I:2)	Female	Dead / 75 y	na	na	na	na	na
1 (II:1)	Male	Dead / 68 y (bladder cancer)	na	na	na	na	na
1 (II:2)	Female	Dead / 73 y (pulmonary cancer)	na	na	na	Yes (pm)	na
1 (II:3)	Male	Dead / 54 y (cancer)	na	na	na	na	na
1 (II:4)	Female	Dead / 61 y (stroke/aneurism)	na	na	na	na	na
1 (II:5)	Female	Asymptomatic / 74 y	320	348	100	Yes	no
1 (II:6)	Male	One syncope young / 70 y	400	425	160	No	no
1 (II:7)	Female	Asymptomatic / 69 y	410	410	200	No	no
1 (II:8)	Female	Syncopes young / 66 y	300	355	120	Yes	yes
1 (II:9)	Male	Asymptomatic / 64 y	360	332	140	Yes	yes
1 (II:10)	Female	Syncopes young / 63 y	300	300	140	Yes	yes
1 (III:1)	Female	Asymptomatic / 54 y	400	393	220	No	no
1 (III:2)	Female	Asymptomatic / 50 y	360	392	160	No	no
1 (III:3)	Male	na	na	na	na	na	na
1 (III:4)	Male	Dead / 41 y (SCD)	na	na	na	na	na
1 (III:5)	Male	Dead / 43 y (SCD)	na	na	na	na	na
1 (III:6)	Female	Asymptomatic / 61 y	400	448	200	No	no
1 (III:7)	Male	Dead / young (traffic accident)	na	na	na	na	na
1 (III:8)	Female	Asymptomatic / 56 y	280	346	100	Yes	yes
1 (III:9)	Female	Asymptomatic /55 y	320	335	140	Yes	yes
1 (III:10)	Female	Asymptomatic / 55 y	400	465	160	No	no
1 (III:11)	Female	Asymptomatic / 53 y	360	407	160	No	no
1 (III:12)	Male	Asymptomatic /50 y	360	360	110	Yes	no
1 (III:13)	Male	Syncope at 27 y / 43 y	300	337	100	Yes	yes
1 (III:14)	Male	Asymptomatic* LVEF 60% / 56 y	300	335	140	Yes	yes
1 (III:15)	Male	Asymptomatic / 54 y	370	380	200	No	no
1 (III:16)	Female	Asymptomatic / 45 y	380	410	160	No	no
1 (III:17)	Male	ACA at 31 y / 39 y	320	320	120	Yes	yes
1 (III:18)	Female	Colitis Ulcerosa / 40 y	320	345	140	Yes	yes
1 (III:19)	Female	Asymptomatic / 40 y	380	380	160	No	no
1 (III:20)	Male	Asymptomatic / 44 y	340	400	160	No	no
1 (III:21)	Female	Asymptomatic / 43 y	360	345	140	Yes	yes
1 (III:22)	Male	Asymptomatic / 40 y	380	380	160	No	no
1 (IV:1)	Male	Severe syncope at 27 y / 36 y	320	320	120	Yes	yes
1 (IV:2)	Female	Asymptomatic / 33	420	420	200	No	no
1 (IV:3)	Male	Asymptomatic / 39 y	320	320	120	Yes	yes
1 (IV:4)	Female	Asymptomatic / 37 y	280	323	100	Yes	yes
1 (IV:5)	Male	Asymptomatic / 35 y	360	386	160	No	no
1 (IV:6)	Male	Asymptomatic / 33 y	380	390	160	No	no

1 (IV:7)	Female	Asymptomatic / 32 y	300	333	120	Yes	no
1 (IV:8)	Male	Asymptomatic / 31 y	320	374	140	No	no
1 (IV:9)	Male	Asymptomatic / 36 y	340	390	160	No	no
1 (IV:10)	Male	Asymptomatic / 34 y	300	320	100	Yes	yes
1 (IV:11)	Female	Asymptomatic / 26 y	300	355	110	Yes	yes
1 (IV:12)	Male	Asymptomatic / 32 y	340	370	120	Yes	no
1 (IV:13)	Male	DCM* LVEF 35% / 30 y	320	367	120	Yes	yes
1 (IV:14)	Male	Asymptomatic / 30 y	390	390	160	No	no
1 (IV:15)	Male	Asymptomatic / 19 y	340	340	120	Yes	no
1 (IV:16)	Male	Asymptomatic / 16 y	340	362	140	Yes	no
1 (IV:17)	Male	Dizziness episodes / 20 y	280	346	120	Yes	yes
1 (IV:18)	Female	Asymptomatic / 18 y	400	400	200	No	no
2 (I:1)	Male	Dead / 92 y	na	na	na	na	na
2 (I:2)	Female	Dead / 78 y	na	na	na	na	na
2 (II:1)	Female	Asymptomatic / 61 y	300	355	140	Yes	no
2 (II:2)	Male	Asymptomatic / 57 y	400	400	240	No	no
2 (II-3)	Female	Asymptomatic / 64 y	360	354	140	Yes	no
2 (II-4)	Male	Asymptomatic / 65 y	340	354	140	Yes	no
2 (III-1)	Male	Dead / 22 y (SCD)	na	na	na	na	na
2 (III-2)	Male	Asymptomatic / 25 y	320	345	100	Yes	yes

ACA = aborted cardiac arrest; DCM = Dilated cardiomyopathy; Jp-Tp = Jpoint-Tpeak interval; LVEF = Left ventricular ejection fraction; na = not available; QTc = Bazett corrected QT interval; SCD = sudden cardiac death; pm = postmortem.

* Heterozygous for MYH7 (NM_000257.2):c.5748G>C, p.Gln1916His

Supplementary Table 2. MOMA NGS Heart panel v1

Gene symbol	Gene name	HGNC ID	Location
ABCC9	ATP-binding cassette, sub-family C (CFTR/MRP), member 9	HGNC:60	12p12.1
ACTA2	actin, alpha 2, smooth muscle, aorta	HGNC:130	10q23.31
ACTC1	actin, alpha, cardiac muscle 1	HGNC:143	15q14
ACTN2	actinin, alpha 2	HGNC:164	1q42-q43
AKAP9	A kinase (PRKA) anchor protein (yotiao) 9	HGNC:379	7q21-q22
ANK2	ankyrin 2, neuronal	HGNC:493	4q25-q26
APOB	apolipoprotein B	HGNC:603	2p24-p23
CACNA1C	calcium channel, voltage-dependent, L type, alpha 1C subunit	HGNC:1390	12p13.3
CACNA1G	calcium channel, voltage-dependent, T type, alpha 1G subunit	HGNC:1394	17q22
CACNB2	calcium channel, voltage-dependent, beta 2 subunit	HGNC:1402	10p12
CALM1	calmodulin 1 (phosphorylase kinase, delta)	HGNC:1442	14q32.11
CALR3	calreticulin 3	HGNC:20407	19p13.11
CASQ2	calsequestrin 2 (cardiac muscle)	HGNC:1513	1p13.1
CAV3	caveolin 3	HGNC:1529	3p25
CBS	cystathionine-beta-synthase	HGNC:1550	21q22.3
CSRP3	cysteine and glycine-rich protein 3 (cardiac LIM protein)	HGNC:2472	11p15.1
CTF1	cardiotrophin 1	HGNC:2499	16p11.2
DES	desmin	HGNC:2770	2q35
DSC2	desmocollin 2	HGNC:3036	18q12.1
DSG2	desmoglein 2	HGNC:3049	18q12.1
DSP	desmoplakin	HGNC:3052	6pter-p21
DTNA	dystrobrevin, alpha	HGNC:3057	18q12
EYA4	eyes absent homolog 4 (Drosophila)	HGNC:3522	6q23
FBN1	fibrillin 1	HGNC:3603	15q21.1
FKTN	fukutin	HGNC:3622	9q31-q33
GJA5	gap junction protein, alpha 5, 40kDa	HGNC:4279	1q21.1
GLA	galactosidase, alpha	HGNC:4296	Xq21.3-q22
GPD1L	glycerol-3-phosphate dehydrogenase 1-like	HGNC:28956	3p22.3
JPH2	junctophilin 2	HGNC:14202	20q12-q13.11
JUP	junction plakoglobin	HGNC:6207	17q21
KCNA5	potassium voltage-gated channel, shaker-related subfamily, member 5	HGNC:6224	12p13
KCNE1	potassium voltage-gated channel, Isk-related family, member 1	HGNC:6240	21q22.1-q22.2
KCNE2	potassium voltage-gated channel, Isk-related family, member 2	HGNC:6242	21q22.1
KCNH2	potassium voltage-gated channel, subfamily H (eag-related), member 2	HGNC:6251	7q36.1
KCNJ2	potassium inwardly-rectifying channel, subfamily J, member 2	HGNC:6263	17q24.3
KCNQ1	potassium voltage-gated channel, KQT-like subfamily, member 1	HGNC:6294	11p15.5
KCNQ4	potassium voltage-gated channel, KQT-like subfamily, member 4	HGNC:6298	1p34

LAMP2	lysosomal-associated membrane protein 2	HGNC:6501	Xq24-q25
LDB3	LIM domain binding 3	HGNC:15710	10q22.3-q23.2
LDLR	low density lipoprotein receptor	HGNC:6547	19p13.2
LMNA	lamin A/C	HGNC:6636	1q22
MTHFR	methylenetetrahydrofolate reductase (NAD(P)H)	HGNC:7436	1p36.3
MYBPC3	myosin binding protein C, cardiac	HGNC:7551	11p11.2
MYH6	myosin, heavy chain 6, cardiac muscle, alpha	HGNC:7576	14q11.2-q13
MYH7	myosin, heavy chain 7, cardiac muscle, beta	HGNC:7577	14q11.2-q13
MYL2	myosin, light chain 2, regulatory, cardiac, slow	HGNC:7583	12q24.11
MYL3	myosin, light chain 3, alkali; ventricular, skeletal, slow	HGNC:7584	3p
MYOZ2	myozenin 2	HGNC:1330	4q26-q27
NEXN	nexilin (F actin binding protein)	HGNC:29557	1p31.1
NPPA	natriuretic peptide A	HGNC:7939	1p36.21
PCSK9	proprotein convertase subtilisin/kexin type 9	HGNC:20001	1p34.1-p32
PKP2	plakophilin 2	HGNC:9024	12p11
PLN	phospholamban	HGNC:9080	6q22.1
PRKAG2	protein kinase, AMP-activated, gamma 2 non-catalytic subunit	HGNC:9386	7q35-q36
RBM20	RNA binding motif protein 20	HGNC:27424	10q25.3
RYR2	ryanodine receptor 2 (cardiac)	HGNC:10484	1q43
SCN1B	sodium channel, voltage-gated, type I, beta subunit	HGNC:10586	19
SCN4B	sodium channel, voltage-gated, type IV, beta subunit	HGNC:10592	11q23.3
SCN5A	sodium channel, voltage-gated, type V, alpha subunit	HGNC:10593	3p21
SGCD	sarcoglycan, delta (35kDa dystrophin-associated glycoprotein)	HGNC:10807	5q33-q34
SLC25A4	solute carrier family 25 (mitochondrial carrier; adenine nucleotide translocator), member 4	HGNC:10990	4q35
SNTA1	syntrophin, alpha 1	HGNC:11167	20q11.2
TAZ	tafazzin	HGNC:11577	Xq28
TCAP	titin-cap	HGNC:11610	17q12
TGFB3	transforming growth factor, beta 3	HGNC:11769	14q24
TGFBR2	transforming growth factor, beta receptor II (70/80kDa)	HGNC:11773	3p22
TMEM43	transmembrane protein 43	HGNC:28472	3p25.1
TMPO	thymopoietin	HGNC:11875	12q22
TNNC1	troponin C type 1 (slow)	HGNC:11943	3p21.1
TNNI3	troponin I type 3 (cardiac)	HGNC:11947	19q13.4
TNNT2	troponin T type 2 (cardiac)	HGNC:11949	1q32
TPM1	tropomyosin 1 (alpha)	HGNC:12010	15q22.1
TTN	titin	HGNC:12403	2q31
TTR	transthyretin	HGNC:12405	18q12.1
VCL	vinculin	HGNC:12665	10q22.1-q23

Supplementary Table 3. Exome sequencing statistics

Family/ relatives	Exome coverage	SLC4A3 c.1109	Position coverage	Frequency SLC4A3 c.1109A	Forward reads SLC4A3 c.1109A	Reverse reads SLC4A3 c.1109A	Average quality SLC4A3 c.1109A
1 (II:6)	117.3	Wild type	179	Wild type	Wild type	Wild type	Wild type
1 (III:6)	102.1	Wild type	139	Wild type	Wild type	Wild type	Wild type
1 (III:16)	116.2	Wild type	196	Wild type	Wild type	Wild type	Wild type
1 (III:17)	105.8	Heterozygous	162	77/162 (47.53%)	24	53	33.16
1 (IV:1)	81.3	Heterozygous	111	46/111 (41.44%)	19	28	33.61
1 (IV:2)	88.8	Wild type	86	Wild type	Wild type	Wild type	Wild type
1 (IV:3)	121.1	Heterozygous	237	126/237 (53.16%)	35	91	33.1
1 (IV:4)	133.6	Heterozygous	220	111/120 (50.45%)	33	78	32.76
2 (I:1)	52.5	Heterozygous	40	15/40 (37.50%)	4	11	37.53
2 (I:2)	68.5	Wild type	86	Wild type	Wild type	Wild type	Wild type
2 (II:2)	86.4	Heterozygous	71	38/71 (53.52 %)	9	29	33.74
